# A randomized controlled study comparing three food prescription programs for older adults: program design, implementation, and utilization

**DOI:** 10.3389/fpubh.2026.1855988

**Published:** 2026-09-24

**Authors:** Lucia A. Leone, Anne Lally Mathiebe, Jennifer Stoll, Rocco A. Paluch, Jill N. Tirabassi

**Affiliations:** 1 Department of Community Health and Health Behavior, University at Buffalo, Buffalo, NY, United States; 2 Department of Family Medicine, Jacobs School of Medicine and Biomedical Sciences, University at Buffalo, Buffalo, NY, United States; 3 Department of Pediatrics, Jacobs School of Medicine and Biomedical Sciences, University at Buffalo, Buffalo, NY, United States

**Keywords:** food prescription, fruit and vegetable consumption, implementation, older adults, usage

## Abstract

**Objective:**

Food prescriptions can help address food insecurity, however older adults have unique barriers to food prescription utilization, including transportation, that threaten program effectiveness. This research compares utilization of two delivered food prescription programs with a standard of care control.

**Design:**

Participants were randomized to be offered one of three 12-week free food prescriptions: (1) Home-Delivered Produce Prescription: a customizable fresh produce box, (2) Home-Delivered Meal Kits: an equivalent amount of produce plus complementary food items to make three recipes, or (3) Mobile Market Produce Prescription (control): a voucher to use at local mobile market. Prescription redemption and produce usage were reported on weekly phone or text surveys.

**Setting:**

Mobile market or home delivery.

**Participants:**

Patients aged 65 and older from hospital-based primary care clinics with difficulty accessing fresh fruits and vegetables (*N* = 67).

**Results:**

There were no statistically significant differences in enrollment rates across arms: 84.6% in produce prescription (*n* = 22), 88.5% in meal kit (*n* = 23), and 73.3% in mobile market (*n* = 22). Self-reported redemption across 12 weeks was higher (*p* < 0.001) in the produce prescription (89.2%) and meal kit (89.2%) arms compared to control (28.3%). Among those who redeemed their prescription, produce usage did not differ, but total possible dosage received was significantly higher (*p* < 0.001) in the produce prescription (75.7%) and meal kit (77.7%) compared to the control (24.9%).

**Conclusion:**

While redemption rates were higher in both delivery arms, the meal kit did not improve produce usage above control, suggesting that delivery had the most impact on food prescription utilization among older adults.

## 1 Introduction

Fruit and vegetable (F&V) consumption is an important component of disease prevention and adults who consume more fresh produce are less likely to develop heart disease, diabetes, certain types of cancer, and are more likely to sustain a healthy weight (1–4). Food insecurity (FI) rates are higher among adults living with chronic diseases including diabetes, hypertension, coronary heart disease and congestive heart failure (5). Concurrent FI may lead to worsened health status and is also associated with higher health care utilization and costs including more emergency room visits and hospitalizations (6, 7). Given that the older adult population is continuing to increase in the U. S. (8) and that FI in older adults continues to rise (9), there is an urgent need to develop FI interventions for this population (8, 10).

Research has shown that food prescriptions can address FI and help prevent and manage disease. A systematic review concluded that food prescriptions may improve F&V consumption and reduce FI, but that more studies with stronger designs are needed (11). Nevertheless, the few studies of produce prescriptions that included comparison groups have shown improvements in F&V consumption and food security (12, 13). However, low food prescription redemptions remain a consistent threat to program effectiveness. In a recent systematic review of food prescription program, five studies reported usage of food voucher redemption rates ranging from 34.5 to 59% with between 63 and 73% of participants redeeming vouchers at least once and between 9 and 18% of participants redeeming all their vouchers (11). Barriers to participation were found at the individual level (cooking skills and tools) and the program level (access to farmers’ markets, produce quality, lack of employee training on voucher redemption, poor communication with participants, and technological issues) (11). Another study found inability to choose desired vegetables or not having enough to share with children or other families were barriers (14). Success was mixed when programs attempted to address transportation issues by facilitating free transportation to farmers’ markets or working with mobile markets (15, 16).

The current study was designed to fill a gap in the food prescription literature, generally, and among older adults specifically. At the time of its development, there were no randomized controlled trials designed to look at usage of different types of food prescription programs. This paper will describe the intervention design, implementation and utilization of three food prescription interventions. This research also responds to previously published recommendations highlighting a need to incorporate more implementation science into Food is Medicine research (17).

## 2 Materials and methods

This paper describes the development, implementation, and usage of three free food prescription interventions. Participants in this study were identified through local medical practices, randomized to an intervention arm and then recruited to that arm by the research team over approximately 2 months. After all participants had been recruited, they started a 12-week intervention period where they received food prescriptions and educational materials. Follow-up data collection took place over approximately 2 weeks after the intervention ended. All methods were approved by the University at Buffalo Institutional Review Board (Study #00008020).

### 2.1 Theory and formative work

Research design for this study followed an iterative process created to ensure that the final interventions were implemented in line with participant needs. To this end, we developed the intervention using theory and formative work with the target population. First, we worked with a Community Advisory Board (CAB) to oversee intervention design. Then we used the Practical, Robust Implementation and Sustainability Model (PRISM) to guide the study and to make iterative changes to intervention delivery to ensure that we are collecting data needed to inform future implementation of Food is Medicine programs. Following this framework, we monitored implementation data over the first 8 weeks of the intervention and adjusted our protocols to ensure optimal implementation.

#### 2.1.1 Behavioral theory

The interventions for this study are based on the behavioral economic principles of “default options” and choice architecture where a “choice architect” sets defaults to create an environment which shifts the path of least resistance to favor a certain behavior (18), in this case redeeming a food prescription. Bundling is used by retailers to encourage purchasing of certain food items by selling them together as one package (19, 20). Behavioral economics indicates that people prefer default options, like bundles, because they are perceived as more convenient and lower in price (20, 21). In the case of a produce prescriptions, bundles reduce the amount of time it would have taken to find each item individually (i.e., search cost) (22, 23). Our previous research with mobile markets has shown that produce bundles may improve purchase and usage of F&V (24). Following behavioral principles of choice architecture, intervention participants in this study automatically received the default food prescription if they did nothing, but had the option to customize their deliveries if they chose to.

#### 2.1.2 Community advisory board

Using convenience sampling, we recruited Community Advisory Board (CAB) members from an urban community center with whom researchers had previously established relationships as part of other studies in older adult populations. We targeted this group because we identified it as representative of the target study population, and the community center was located 2.5 miles from the clinical partner. We conducted two in-person presentations during which study team members provided a flier, an overview of the research project, and details on member commitment. Interested individuals signed an interest sheet and were later contacted by a study team member over the phone for eligibility screening. Eligibility criteria included being aged 65 + and/or a caregiver to someone aged 65+. Further eligibility criteria required participants to have the ability to store and prepare meals in their homes. Sixteen members signed the interest form, and 14 members were recruited for the CAB. At least two team members facilitated the meetings and provided light refreshments and gift cards for $50 per session. CAB meeting topics and discussion points can be found in Table 1. In addition to planning for the intervention, all CAB members received 1 week of meal kits and were asked to place their meal kit orders and customize them through the food partner website.

**Table 1:** Overview of Community Advisory Board (CAB) process and major findings from meetings held between May 2024 and August 2024 to inform a food prescription program for older adults in Buffalo, NY.

Intervention development steps	Discussion and feedback
CAB meeting 1: program overview	Overview of study Discussion of general food considerations in older adults (e.g., chewing and dietary restrictions) Discussion of possible recipes and study adaptations such as feasibility of preparation by family members or caretakers
CAB meeting 2: discuss education and recipes with nutritionist	Discussion of participant’s cooking skills, eating patterns and equipment needs Feedback on potential educational formats and delivery methods
CAB meeting 3: discussion of technology	Review of the online ordering platform and discussion of support needed to use it including the need for a call-in option Discussion of preferred technology use and frequency of study and ordering communications (i.e., text, phone call, email)
CAB meeting 4: discussion of communications and initial meal kit delivery	CAB members reviewed the research study consent form and recruitment materials (intervention descriptions) and provided suggestions to improve clarity Feedback on appearance of food and recipes for the meal kits
CAB meeting 5: review of meal kits and recipes	Discussion of CAB member experiences with meal kits they received, and any issues related to meal kit contents and recipes Feedback on types of recipes and adherence to recipes in preparation of meals; CAB members requested more adventurous or “ethnic” recipes as they already were experienced at cooking and did not need simple recipes to use the food
Survey testing: phone calls with CAB members	CAB members pre-tested the baseline survey for length, comprehension and content and suggested removal of certain scales that seemed unrelated to the study and could cause distress among participants (e.g., loneliness scale, questions on mortality)

### 2.2 Intervention design

This was a three-arm randomized controlled trial with two intervention arms and a usual care (Table 2). The intervention arms included home-delivered produce prescriptions and home-delivered meal kits. The control participants received a voucher to receive an equivalent amount of produce as the intervention arms at a mobile market (approximately 12 servings/week per person). Participants in all arms also received cooking utensils and equipment (e.g., pots, pans, knives). The intervention arms for this study used a default subscription model where participants can “set it and forget it” or choose to customize their food every week. Both intervention arms were designed using behavioral economic principles of default bias and used home delivery to reduce stigma and transportation barriers as well as improve participant choice. *We hypothesized that both intervention arms would increase food prescription redemption above the usual care control arm*, which mirrors typical produce prescription programs in our region and nationwide. Additionally, the healthy meal kit arm provided all the ingredients needed to make 3 meals/week as well as instructions designed to increase cooking self-efficacy. *Thus, we hypothesized that meal kits would also increase F&V usage above control arm.*

**Table 2:** Summary of intervention arms and characteristics of a food prescription program serving older adults in Buffalo, NY from September 2024 to January 2025.

Study arm	Default food provided	Preference accommodated	Customization	Accessibility	Recipes/food education
Mobile market produce prescription (usual care control)	12 servings of fruits and vegetables per person	Family size	Choose any produce available at market	10 + weekly market locations across city	Sent all recipes and educational materials via mail at beginning of study
Delivered produce prescription	12 servings of fruits and vegetables per person	Family size and dietary preferences	Selection of produce is fully customizable	Home delivery	Sent all recipes via mail at beginning of study. Weekly education in delivery box
Delivered meal kit	All ingredients to make 3 + meals with 12 servings of fruits and vegetables per person	Family size and dietary preferences	Choose from 5–6 meal options each week	Home delivery	Meal specific recipes and education provided weekly in delivery box

#### 2.2.1 Mobile market produce prescription (standard of care control)

Mobile market participants received a weekly voucher to purchase produce boxes (or an equivalent amount of produce) from a mobile market at the medical center from which participants were recruited or from any of the 11 mobile market locations run by one of our community partners. Participants were asked to choose the market at which they would shop at the beginning of the study so that our mobile market partners could ensure they had food available for their full prescription, but the voucher could be redeemed at any participating market. Given the limited traffic at the medical center, produce boxes (the same as provided in delivery arm) were purchased in advance and delivered to the mobile market for pick-up by participants. Participants could accept these boxes, customize them, or shop for other items at the market. Participants received all 12 of their weekly vouchers via mail at the beginning of the study but each one was only available for use on a designated week.

#### 2.2.2 Delivered produce prescription

Produce prescription participants received weekly home deliveries of fresh F&V. Produce prescriptions were packed and delivered by a local business that aggregates food from local farms and delivers to homes. Produce box contents changed weekly based on seasonal availability, but the amount of food given weekly remained constant throughout the study. At enrollment, participants were signed up to receive a weekly box based on their household size (small box for 1–3 or large box for 4–6 people). These boxes were chosen as they were standard sizes already offered by the delivery partner and would facilitate easier program implementation and ensure participants had at least the minimum amount of food required for their prescription. Participants had the ability to fully customize their produce selections each week through the online ordering platform or by calling a study coordinator. The customization window offered by the delivery partner was between Friday afternoon and Sunday at midnight. The study coordinator was available to take phone orders between 2 and 4 p.m. on Fridays. Participants could also skip their delivery, however, if they did nothing, they received a default box.

#### 2.2.3 Delivered meal kit

Meal kit participants received all the ingredients each week to make three recipes. Meal kits were designed by a study dietician with feedback from the CAB and our food partners. Recipes were selected to be compliant with common healthy diets and with attention to balancing macronutrients and including low glycemic index foods in order to make them diabetic friendly. The amount of produce in the meals was designed to be in line with the produce prescription condition. Recipes were provided to the food partner who sourced and packed the food for the recipes. Food in the meal kits was portioned when possible, however participants also received a stock-up box of common ingredients (e.g., olive oil, vinegar, spices) at the beginning of the study to limit the need for portioning of frequently used items. Meal kits were designed to have either two or four servings each (depending on household size) and participants had the option to choose three meal kits each week from a menu of 5 or 6 options. Weekly options were repeated every 4 weeks and at the beginning of each 4-week period participants could choose their meal kit choices for the following 4 weeks either by phone or e-mail. This was done to make it easier for the participants and allow the food provider to plan food purchases in advance. Those who did not choose their meals received three default options with accommodation for vegetarian diets. In order to have an equivalent amount of produce as the two produce prescription arms, participants also received a selection of fruit each week which was not part of the meals themselves. Participants in this arm also had the option to skip their delivery either online or by phone.

#### 2.2.4 Participant communications and education

In both intervention arms, participants received weekly printed information sheets with food and nutrition education inside of their boxes. In addition, all arms received the meal kit recipes; they were sent weekly (with the corresponding ingredients) in the meal kit arm and at the beginning of the study in the other arms. Participants in the intervention arms who provided an e-mail or phone number received alerts when their orders could be customized as well as delivery reminders. Mobile market participants were sent text reminders to visit the market prior to their chosen market day. Participants were sent a weekly process survey (described below in measures) by text or email (based on their preference) on the evening of their food delivery day (Wednesday or Thursday). The survey also collected data on any challenges that participants may have had with their food prescription, including prescription redemption, food usage, and survey completion. These responses were reviewed by the study coordinator each week and any problems with delivery or food received were conveyed to the food partners and issues were addressed as they would be with traditional customers. If necessary, the study coordinator made follow-up calls to the participants to troubleshoot their issues. If intervention participants did not have any contact with the study team for 2 weeks (i.e., no response to surveys or e-mails, no online login, or no phone calls), their deliveries were discontinued until they reinitiated contact.

### 2.3 Eligibility, randomization, recruitment and Enrollment

#### 2.3.1 Participant identification

We worked with two primary care clinics of a local hospital system to identify and recruit eligible participants. Eligible participants were patients aged 65 and older who screened positive for FI through an existing two question screener that all patients receive at their annual visit. This screener asks patients if “in the last 12 months, did you ever, eat less than you felt you should because there wasn’t enough money for food” and “do you have access to fresh fruits and vegetables within two miles of your residence.” Patients who answered in the affirmative for the first or negative to the second question were eligible for the study. The population health team extracted an initial list of potential participants from the medical records of patients who visited the included clinics in the past year. All potentially eligible participants received a mailed information sheet about the study and a letter from the primary care clinic encouraging them to participate. The letter stated they had 2 weeks to opt out of being contacted by a research team member from the University at Buffalo for potential recruitment. Then, the contact information for those who did not opt-out was provided to the research team. In addition to the initial list, some participants were recruited after viewing a flyer at their healthcare provider’s office and contacting the research team directly or being referred to the study by their physician.

#### 2.3.2 Randomization and recruitment

As enrollment rate was an outcome for this study, we wanted to understand if there would be differential enrollment based on the intervention which participants were offered. Thus, randomization occurred prior to recruitment and participants were unaware of the unoffered intervention arms to avoid introducing biases due to comparison (i.e., the consent form only described their arm). Randomization for the study was designed following the blocked randomization with randomly selected block sizes method described by Efird (25) to increase the probability of even distribution of our finite pool of potential participants across interventions. We randomized participant slots within blocks of random size, using separate randomization tables for male and female participants to ensure even distribution of genders across all arms. Each randomized slot remained open until filled with an eligible participant; if an individual was not interested in the arm they were offered at recruitment, the slot for that arm remained open. Therefore, each arm was populated with participants evenly as we worked through our recruitment list. This process, coupled with blinding the unoffered intervention arms, allowed us to measure which intervention arm had the highest enrollment rate based on how many attempts it took to reach comparable enrollment for each arm. The research team called potential participants who did not opt out of being contacted. The potential participant was recruited to the specific intervention arm slot that was currently being filled without describing the two additional intervention arms of the study (See Supplementary Table 1 for descriptions). If they were interested, we further assessed eligibility based on the following criteria: English speaking, age 65 or older, ability to store food and prepare meals at home, being the primary grocery shopper for their household, having no severe food allergies, and receiving care through our clinical partner. If the individual was not the primary grocery shopper, their spouse or caregiver was considered for participation (in addition to the patient), provided they met the other eligibility requirements, and they joined the study as a pair.

#### 2.3.3 Enrollment

After recruitment, participants were verbally consented and scheduled to complete a baseline survey over the phone. After the baseline survey was completed, they received a packet in the mail detailing study activities including timeline, consent form, and intervention arm-specific information such as participating mobile market locations, meal kit selections, and account information to login and customize their produce box using an online platform. Finally, they completed a phone call with a research coordinator to orient them to the study arm to which they were randomized and troubleshoot any issues they may have redeeming their prescriptions. During this call, participants were assessed to determine which cooking supplies they would need (from a list of 23 common kitchen tools) in order to be able to use the food provided. Once enrolled, participants started the intervention to which they were assigned and received 12 deliveries over 15 weeks with three break weeks for public holidays including Thanksgiving, Christmas, and New Year’s Day. Program enrollment was measured as the percentage of eligible referred patients that decided to enroll in the program they were offered. Patients who chose not to enroll were asked to provide more information on why they did not want to enroll.

### 2.4 Data collection and measures

Participant data was collected using three difference instruments: (1) a baseline phone survey after enrollment, but before the intervention started; (2) a weekly process survey during each week of the intervention starting on the second week, and (3) a follow-up phone survey administered 1–2 weeks after the intervention ended. If a potential participant reported not completing grocery shopping for the household, they were asked if their spouse or other household member that completes the majority of food shopping for the household would be eligible and interested in participating; in the case of a paired enrollment, only one process measure survey was sent per household and was completed by the primary shopper, but both participants completed baseline and follow-up surveys.

#### 2.4.1 Participant demographics

All demographics were collected as part of the baseline survey. Gender was self-reported using an open-ended survey question, with the interviewer then assigning the response to a predefined category (man, woman, non-binary/third gender, prefer to self-describe, prefer not to answer). Racial and ethnic identification were also self-reported using the convention established by the 2020 United States Census. Additional demographics included age, household income, marital status, education, employment status, government assistance participation in the past 12 months (e.g., Supplemental Nutrition Assistance Program [SNAP], Medicaid, etc.) and total mouths to feed within the household (children and adults). Eligible spouses/caregivers were eligible to complete individual baseline and follow-up data in addition to the patient.

#### 2.4.2 Program utilization measures

Redemption was measured using a question in the weekly process measure survey that asked participants to confirm prescription redemption (receipt of delivery). Usual care control arm participants also received weekly surveys asking if they redeemed their prescription at the mobile market and how much food they used. The final week of utilization data was collected during the follow-up survey in lieu of another weekly survey. To determine redemption, we calculated the percentage of all 12 deliveries/mobile market visits that were confirmed via the weekly survey. We also calculated the average number of prescriptions redeemed in each study arm. If a survey was not completed for a given week, that data was considered missing. Redemption was confirmed by reviewing delivery partner records. For the mobile market condition, we had the partner return vouchers that were collected from participants. Self-report data was used as our main outcome for consistency of analyses.

Usage was measured in the self-reported weekly process surveys using a question that asked participants approximately what percentage of the F&Vs that they received were used by someone in their household: all (coded as 100%), most (coded as 75%), about half (coded as 50%), some (coded as 25%) and none (coded as 0%). Answers from all completed surveys over 12 weeks were averaged to calculate usage for each participant. Weeks in which a participant reported not redeeming a prescription were coded as missing for the purpose of this variable.

Total Dosage was calculated as the percentage of all possible food that participants could receive during the intervention that they reported using. This was a combination of both redemption and usage. For example, if a participant reported on average redeeming 50% of their prescriptions and reported using 75% of the food they received, their total dosage would be 37.5% (75% x 50%). If a participant did not report redeeming a prescription or their data was missing for a given week, they were considered to not have any dosage received for that week.

*Customization and recipe preparation*: All participants were asked how many study-provided recipes were prepared on average across all weeks and the main reasons for not using study recipes. For the meal kit group, we looked at percent of recipes made for the meal kits (out of a total possible 36 recipes—3 per week across 12 weeks). Participants in the intervention arms were asked if they customized their orders and those who reported not customizing their orders were asked the main reasons that they did not.

#### 2.4.3 Study perceptions

The follow-up survey included questions about program satisfaction, customization, and perceptions of change in F&V purchasing/eating. For program satisfaction, participants were asked: “how likely is it that you would recommend the Golden Harvest Food Produce Prescription program to a friend or family member? Please answer on a scale of 0-10 with 0 meaning not recommend at all and 10 means you would very highly recommend it.” To gage perceptions of changes in F&V purchasing/eating, participants were asked: “Has the food prescription program changed the amount of fruits and vegetables that your family buys/eats?” Possible answer choices included we are eating/buying more, about the same or less.

### 2.5 Power calculation

Power for this study was based on our primary outcome of food prescription redemption. In order to detect a difference in usage of 45% using a chi-square (*w* = 0.426 at *p* = 0.05 and 80% power) between either intervention arm and the usual care arm, we needed 54 participants (18 per arm). This assumed a redemption rate of 30% for usual care and 75% for delivery. These redemption numbers were conservative compared to published usage rates for usual care (under 18%) (11) and delivery (89%) (26).

### 2.6 Analyses

Enrollment outcomes were compared using frequency tables and chi-square analyses. Descriptive statistics are presented for all participant characteristics and the customization outcome. All other utilization outcomes and study perceptions outcomes were analyzed using Analysis of Variance (ANOVA) and were followed with orthogonal contrasts comparing three combinations of group comparisons (Meal Kit/Produce Prescription, Meal Kit/Mobile Market, Produce Prescription/Mobile Market). A sensitivity analysis also looked at changes in self-reported F&V purchasing and eating as dichotomous outcome to test the hypothesis that the intervention arms reduced purchasing (spent less vs. same or more) or increased intake (ate more vs. same or less). All analyses were conducted using SAS 9.4.

## 3 Results

### 3.1 Participant identification, recruitment and Enrollment

Recruitment, enrollment, and retention data are displayed in Figure 1. Of the patients who received an opt-out letter only three (2.1%) opted-out. An additional 26 (out of 126 reached for recruitment) declined participation prior to hearing the program description (20.6%). In total, 100 patients were randomized to be offered one of the study arms. As there was one spouse included in the study, the total available for recruitment was 101, but the spouse was paired with the patient in the same intervention arm as described above and not randomized. The mobile market arm was offered to 41 potential participants; 22 enrolled, 11 were ineligible and eight were not interested. Of those eight, two cited a lack of transportation/lack of desire to travel to the available markets and six gave no reason. The produce prescription arm was offered to 30 potential participants; 22 enrolled, four were ineligible and four were not interested with no specific reason given. Finally, the meal kit arm was offered to 30 potential participants: 23 enrolled, four were not eligible and three were not interested, two of whom stated that they receive enough food via other programs (e.g., SNAP and food prescription offered through their health insurance). In summary, across all 67 participants, there were no statistically significant differences (*p* = 0.31) in enrollment rates of eligible participants between arms: rates were 84.6% for the produce prescription arm (*n* = 22), 88.5% for meal kit arm (*n* = 23), and 73.3% for the mobile market arm (*n* = 22).

**Figure 1:**
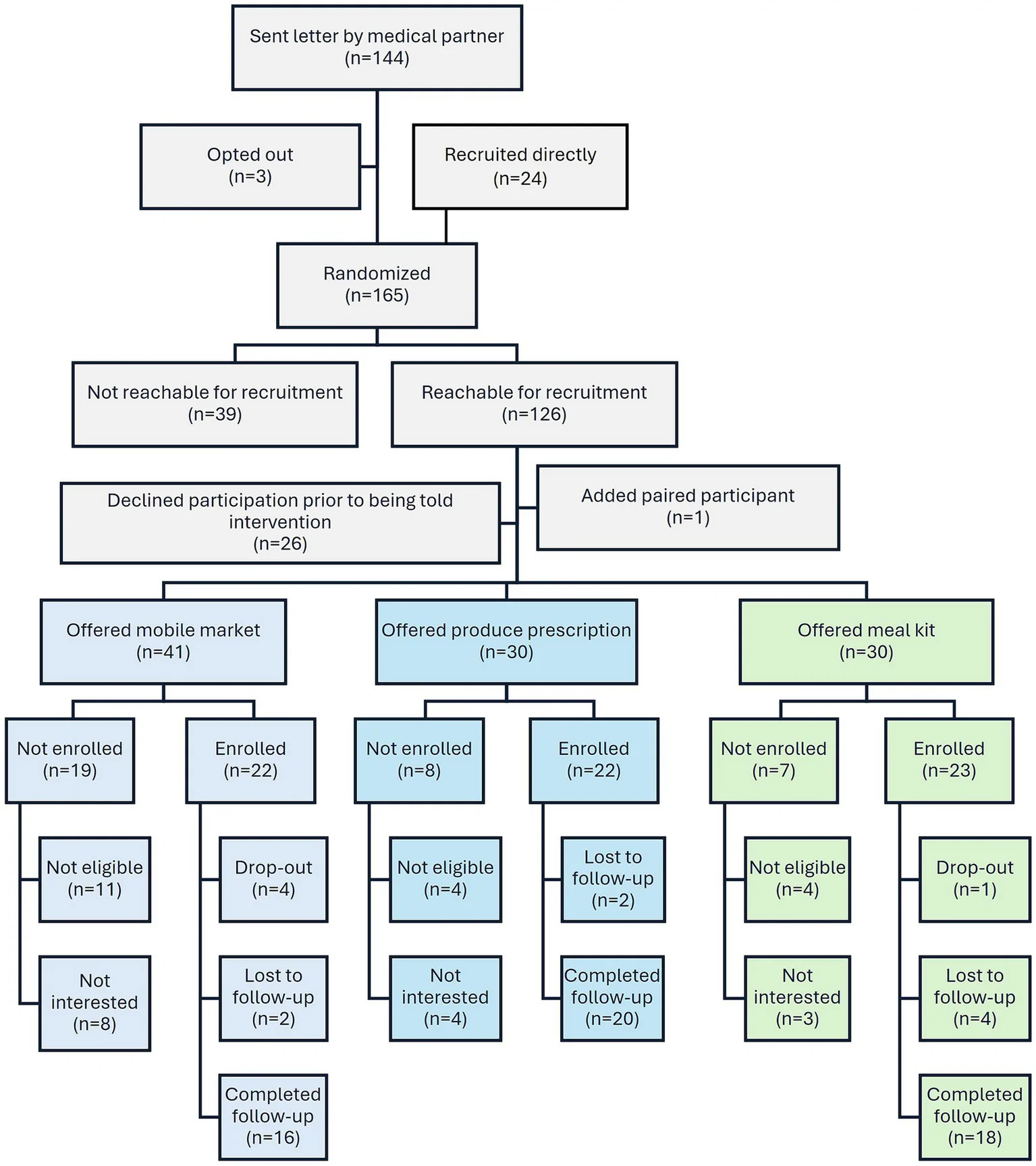
Recruitment and retention of all participants in a food prescription program serving older adults in Buffalo, NY from September 2024 to January 2025. Across all arms, 19 potential participants were ineligible due to not speaking English (7), age (1), inability to store or prepare food (5), not the primary shopper (4), severe allergy (4), and other (3) which included a recent medical emergency/decline. There were 4 people who had multiple reasons for being ineligible. One participant was added to the study during recruitment calls, as the one potential patient was deemed ineligible during screening due to not being responsible for the majority of food shopping and preparation in the household, but reported that his wife was. Upon screening his wife, it was determined that she could enter the study as a paired participant.

### 3.2 Participant characteristics

Participant demographics are shown in Table 3. On average, our sample was 70.9 (SD 5.5) years old and lived in a household with 1.6 adults (SD 1.3) and 0.1 children (SD 0.6). The sample was 53.7% (*n* = 36) female and predominately African American (73.1%, *n* = 49). The majority of participants (55.2%, *n* = 37) reported an annual household income under $20,000 per year. Most participants (53.7%, *n* = 36) reported completing at least some college and being retired (62.7%, *n* = 42). The most common marital status was single (50.8%, *n* = 34) and the most common health insurance was Medicare (41.8%, *n* = 28), though 35.8% (*n* = 24) of participants reporting having multiple insurers. The majority of participants (76.1%, *n* = 51) reported receiving some sort of government or food assistance in the past year (i.e., Medicaid, SNAP, disability benefits, or using a food pantry). More than half (52.7%, *n* = 34) of participants were food insecure.

**Table 3:** Participant demographics for the entire sample and by study arm for a food prescription program serving older adults in Buffalo, NY from September 2024 to January 2025.

Variable	All participants	Mobile market (*n* = 22)	Delivered produce prescription (*n* = 22)	Delivered meal kit (*n* = 23)
Age, Mean (sd)	70.9 (5.5)	70.3 (4.1)	70.1 (5.0)	72.2 (6.9)
Gender, *n* (%)				
Female	36 (53.7%)	12 (54.6%)	12 (54.6%)	12 (52.2%)
Male	30 (44.8%)	10 (45.5%)	9 (40.9%)	11 (47.8%)
Missing/Refused	1 (1.5%)	0 (0%)	1 (4.6%)	0 (0%)
Race *n* (%)				
African American	49 (73.1%)	16 (72.7%)	20 (90.9%)	13 (56.5%)
Pacific Islander	1 (1.5%)	1 (4.6%)	0 (0%)	0 (0%)
White	12 (17.9%)	2 (9.1%)	2 (9.1%)	8 (34.8%)
Multiracial	2 (3.0%)	1 (4.6%)	0 (0%)	1 (4.4%)
Missing/Refused	3 (3.0%)	2 (9.1%)	0 (0%)	1 (4.4%)
Ethnicity, *n* (%)				
Hispanic	1 (1.5%)	1 (4.6%)	0 (0%)	0 (0%)
Non-Hispanic	65 (97.0%)	21 (95.5%)	21 (95.5%)	23 (100%)
Missing/Refused	1 (1.5%)	0 (0%)	1 (4.6%)	0 (0%)
Income, *n* (%)				
<$10,000	21 (31.3%)	6 (27.3%)	8 (36.4%)	7 (30.4%)
$10,000 to $19,999	16 (23.9%)	3 (13.6%)	9 (40.9%)	4 (17.4%)
$20,000 to $29,999	10 (14.9%)	2 (9.1%)	2 (9.1%)	6 (26.1%)
$30,000 to $39,999	5 (7.5%)	3 (13.6%)	1 (4.6%)	1 (4.4%)
$40,000 to $49,999	3 (4.5%)	2 (9.1%)	0 (0%)	1 (4.4%)
$50,000 to $59,999	2 (3.0%)	2 (9.1%)	0 (0%)	0 (0%)
= > $60,000	1 (1.5%)	0 (0%)	1 (4.6%)	0 (0%)
Missing/Refused	9 (13.4%)	4 (18.2%)	1 (4.6%)	4 (17.4%)
Education, *n* (%)				
8th Grade or less	0 (0.0%)	0 (0%)	0 (0%)	0 (0%)
Some high school	8 (11.9%)	3 (13.6%)	2 (9.1%)	3 (13.0%)
High school graduate or GED	18 (26.9%)	4 (18.2%)	6 (27.3%)	8 (34.8%)
Trade/beauty school graduate	5 (7.5%)	0 (0%)	3 (13.6%)	2 (8.7%)
Some college	22 (32.8%)	10 (45.5%)	8 (36.4%)	4 (17.4%)
College graduate	10 (14.9%)	3 (13.6%)	2 (9.1%)	5 (21.7%)
Some postgraduate, post graduate	4 (6.0%)	2 (9.1%)	1 (4.6%)	1 (4.4%)
Missing/Refused	0 (0.0%)	0 (0%)	0 (0%)	0 (0%)
Marital status *n* (%)				
Married	6 (9.0%)	3 (13.6%)	1 (4.6%)	2 (8.7%)
Single	34 (50.8%)	8 (36.4%)	12 (54.6%)	14 (60.9%)
Divorced	8 (11.9%)	4 (18.2%)	3 (13.6%)	1 (4.4%)
Separated	4 (6.0%)	2 (9.1%)	2 (9.1%)	0 (0%)
Widowed	15 (22.4%)	5 (22.7%)	4 (18.2%)	6 (26.1%)
Employment status *n* (%)				
Work full-time	4 (6.0%)	1 (4.6%)	2 (9.1%)	1 (4.4%)
Work part-time	3 (4.5%)	1 (4.6%)	1 (4.6%)	1 (4.4%)
Unemployed	5 (7.5%)	2 (9.09%)	1 (4.6%)	2 (8.7%)
Retired	42 (62.7%)	14 (63.6%)	13 (59.1%)	15 (65.2%)
SSI/Disability	13 (19.4%)	4 (18.2%)	5 (22.7%)	4 (17.4%)
Health insurance *n* (%)				
Employer (own policy)	4 (6.0%)	3 (13.6%)	1 (4.6%)	0 (0%)
Purchased	3 (4.5%)	0 (0%)	0 (0%)	3 (13.0%)
Medicare	28 (41.8%)	8 (36.4%)	8 (36.4%)	12 (52.2%)
Medicaid	5 (7.5%)	1 (4.6%)	2 (9.1%)	2 (8.7%)
Other government plan	2 (3.0%)	2 (9.1%)	0 (0%)	0 (0%)
Multiple policies	24 (35.8%)	8 (36.4%)	10 (45.5%)	6 (26.1%)
Uninsured	1 (1.5%)	0 (0%)	1 (4.6%)	0 (0%)
Govt assistance *n* (%)				
Disability/social security	31 (46.3%)	11 (50.0%)	12 (54.6%)	8 (34.8%)
Food pantry	4 (6.0%)	2 (9.1%)	0 (0%)	2 (8.7%)
Medicaid	9 (13.4%)	2 (9.1%)	3 (13.6%)	4 (17.4%)
SNAP	4 (6.0%)	2 (9.1%)	1 (4.6%)	1(4.4%)
No Govt. Assistance	16 (23.9%)	5 (22.7%)	5 (22.7%)	6 (26.1%)
Missing/refused	3 (4.5%)	0 (0%)	1 (4.6%)	2 (8.7%)
Number of adults, mean (SD)	1.6 (1.3)	1.7 (0.8)	1.9 (2.0)	1.2 (0.4)
Number of children, mean (SD)	0.1 (0.6)	0.3 (0.8)	0.2 (0.7)	0 (0)
Food security, *n* (%)				
High food security	21 (31.3%)	9 (40.9%)	7 (31.8%)	5 (21.7%)
Marginal food security	12 (17.9%)	3 (13.6%)	4 (18.2%)	5 (21.7%)
Low food security	12 (17.9%)	6 (27.3%)	3 (13.6%)	3 (13.0%)
Very low food security	22 (32.8%)	4 (18.2%)	8 (36.4%)	10 (43.5%)

### 3.3 Program utilization

There were differences in food prescription redemptions across all arms (*F* = 42.21, *p* < 0001) (Table 4). Prescription redemptions rates were 89.2% in both intervention arms and 28.3% in the mobile market control arm (Figure 2). Participants in the mobile market arm reported redeeming on average 3.4 (SD 3.4) their prescriptions across 12 weeks (Table 4). Redemption in both intervention arms was greater than in the control arm: 10.7 (SD 2.9) for the delivered produce prescription (*p* < 0.0001) and 10.7 (SD 2.8) for the delivered meal kit arm (*p* < 0.0001). Delivery partner records confirmed that on average across all participants (including drop-outs), 11.1 deliveries were made in the produce prescription arm and 11.3 in the meal kit arm; an average of 1.8 mobile market pick-ups were also confirmed.

**Table 4:** Differences in food prescription utilization overall and by study arm among older adults in Buffalo, NY from September 2024 to January 2025.

Study arm	Mean (SD)	Omnibus *F*-Value (*p*-value)	Contrast	Contrast *F* value (*p*-value)
Redemptions (Number of Weeks)				
Meal Kit	10.7 (2.8)	42.21 (*p* < 0.0001)	Meal Kit vs. Produce Rx	0.01 (*p* = 0.934)
Mobile Market	3.4 (3.4)		Meal Kit vs. Mobile Market	63.89 (p < 0.0001)
Produce Prescription	10.7 (2.9)		Mobile Market vs. Produce Rx	63.81 (p < 0.0001)
Food usage (Percentage of Redeemed)				
Meal kit	85.4 (14.2)	0.12 (*p* = 0.863)	Meal Kit vs. Produce Rx	0.11 (*p* = 0.740)
Mobile market	82.6 (11.1)		Meal Kit vs. Mobile Market	0.06 (*p* = 0.810)
Produce prescription	84.3 (16.6)		Mobile Market vs. Produce Rx	0.29 (*p* = 0.589)
Dosage received (Percentage of total possible)				
Meal kit	77.7 (25.7)	28.96 (*p* < 0.001)	Meal Kit vs. Produce Rx	42.11 (*p* < 0.0001)
Mobile market	24.9 (23.9)		Meal Kit vs. Mobile Market	0.07 (*p* = 0.798)
Produce prescription	75.7 (26.3)		Mobile Market vs. Produce Rx	46.35 (<0.0001)

**Figure 2:**
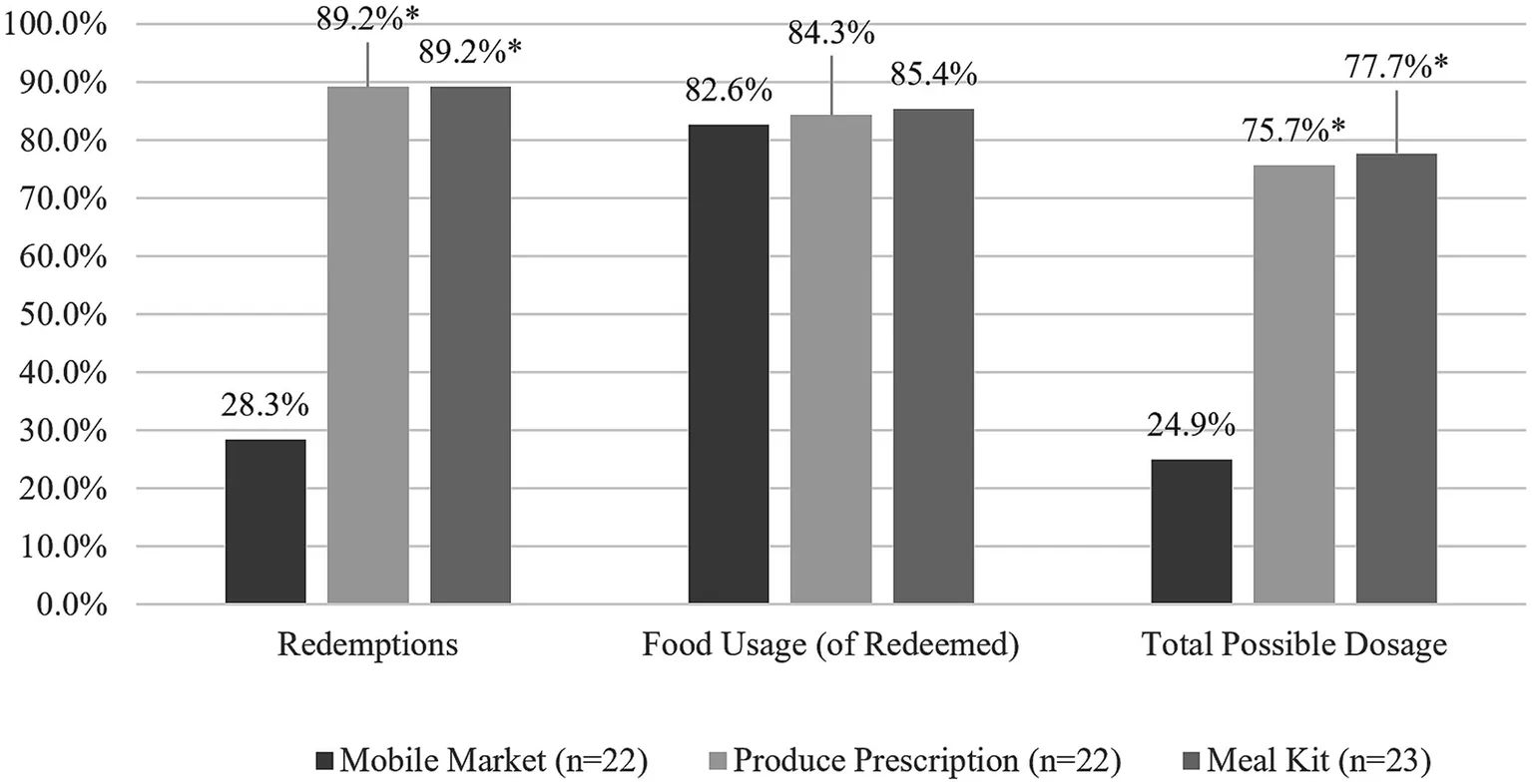
Food prescription utilization across study arms in a food prescription program serving older adults in Buffalo, NY from September 2024 to January 2025 (*n* = 67). *Significantly different than the mobile market (usual care control) arm (*p* < 0.001).

Among participants who reported redeeming their prescriptions, there were no statistically significant differences in reported food usage arms (*F* = 0.12, *p* = 0.863) (Table 4). The mobile market arm reported using 82.6% (SD 11.1%) of their food, delivered produce prescription arm used 84.3% (SD 16.6%) and the meal kit arm used 85.4% (SD 14.2%) of their food across 12 weeks. There were differences in food dosage received across all arms (*F* = 29.96, *p* < 0.001) (Table 4). Participants in the mobile market arm reported using 24.9% (SD 23.9%) of the possible dosage. Dosage received was greater in both intervention arms than in the control arm: 75.7% (SD 26.3%) for the delivered produce prescription (*p* < 0.001) and 77.7% (25.7% SD) for the meal kit arm (*p* < 0.001).

Reported preparation of recipes was higher in both intervention arms (*p* < 0.001); mobile market participants reported using 2.1 (SD 2.8) recipes on average across 12 weeks, delivered produce prescriptions used 4.6 (SD 7.8) recipes and meal kit participants used 26.7 (SD 11.4) recipes. Thus, on average, meal kit participants made 74.1% of the meal kit recipes provided. The main reason for not using recipes (across groups) was lack of time/energy. The second most commonly reported reason in the mobile market and produce prescription groups was not having the ingredients needed to make them. We also asked participants in the intervention arms about customization, and few reported customizing their orders: 7 in the produce prescription arm (5 online and 2 by phone) and 11 in the meal kit arm (1 online and 10 by phone). No one reported issues with the website as a reason for not customizing, 4 participants in the meal group reported they did not have internet access and one person in each group said they were unaware of the option to customize.

### 3.4 Study perceptions

There were no statistically significant differences in mean program satisfaction ratings for intervention vs. control groups: 9.5 (SD 1.4) for the mobile market arm; 9.7 (SD 0.8) for the produce prescription arm and 9.9 (0.5 SD) out of 10 for the meal kit arm (Table 5). At follow-up, we found no statistically significant differences across groups in self-reported change in purchasing of F&*Vs* (Table 5). In the sensitivity analysis we found that participants in the delivered produce prescription arm were more likely (70.6%) than the control group (29.4%) to report that they spent less of their own money on F&V as a result of being in the food prescription program (*p* = 0.042). Overall, more than half of participants agreed that they were eating more F&*Vs* due to the food prescriptions, but there were no statistically significant differences by arm (Table 4).

**Table 5:** Perceptions of how the interventions affected fruit and vegetable related behaviors in a food prescription program serving older adults in Buffalo, NY from September 2024 to January 2025.

Participant response	Mobile market produce prescription arm (*n* = 22)	Delivered produce prescription arm (*n* = 22)	*p*-value (vs. control)*	Delivered meal kit arm (*n* = 23)	*p*-value (vs. Control)*
Perception of change in fruits and vegetables purchased with participants own money due to food prescription, *n* (%)					
More	4 (18.2%)	2 (9.1%)	*p* = 0.13	4 (17.4%)	*p* = 0.37
Same	8 (36.4%)	5 (22.7%)		7 (30.4%)	
Less	5 (22.7%)	12 (54.6%)		7 (30.4%)	
Missing/Refused	5 (22.7%)	3 (13.6%)		5 (21.7%)	
Perception of change in fruits and vegetables eaten due to food prescription					
More	9 (40.9%)	8 (36.4%)	*p* = 1.0	8 (34.8%)	*p* = 0.53
Same	7 (31.8%)	8 (36.4%)		5 (21.7%)	
Less	0 (0%)	0 (0%)		1 (4.4%)	
Missing/Refused	6 (27.3%)	6 (27.3%)		9 (39.1%)	

*Fisher Exact Test to test the differences in frequency of responses between each intervention arm and the control with missing participants excluded.

## 4 Discussion

The goal of this study was to compare utilization of food prescriptions across three different interventions: a delivered meal kit, a delivered produce prescription, and a mobile market-based produce prescription. As hypothesized, we found that prescription redemptions were higher in the delivery arms than in the mobile market control group. We did not find that the additional food provided to make recipes in the meal kit arm led to higher reported F&V usage than in the control. Notably, enrollment and usage of F&*Vs* received was similar and high (over 80%) in all arms indicating that the biggest limitation to food prescription utilization among older adults is getting the food into the home.

Participants in the produce prescription condition, but not the meal kit condition, reported saving money on F&Vs. Similar F&V dosage received in both intervention arms coupled with the cost savings to participants would suggest that the produce prescription intervention is more cost effective for increasing F&V intake, On average the meal kits cost $91–$107 per household/week (delivery included) and the produce prescriptions cost approximately $27–$36 per participant/week (delivery included). The mobile market prescriptions ranged from $14–$22 per participant/week, however the study paid an additional $10,000 to one of the mobile market partners to run the market at the clinic which brings the average to $52–$60 per participant/week. Feedback from the CAB indicated that most of the older adults knew how to cook and were able to use the produce even if they were not given specific recipes and ingredients to make them. Together these findings suggest that the additional cost of meal kits may not be valuable to increasing F&V usage in this population, however future analyses will explore their impact on other health related outcomes.

Redemption rates from this study are in line with another food delivery study with pregnant mothers that found that 87% of participants confirmed that their delivery was received (26); in our study, redemption was reported at 89.2%. While redemption is not frequently reported in the literature, one 6-month food prescription study among adults who redeemed food prescriptions at a food pantry had redemption rates between 35.4 to 39.4% (27). On average, participants redeemed 6.5 of the available 12 redemptions. This was a higher redemption rate than in our mobile market control arm (28.3%), suggesting that older adults may have greater barriers to prescription redemption outside of their homes.

### 4.1 Strengths and limitations

This study has several strengths including using a CAB to support research development and a focus on implementation using PRISM. The CAB provided extensive feedback on how we could shape intervention implementation, communication, and data collection to ensure utilization by the target population. Further, the PRISM framework guided iterative changes to the implementation of the intervention in the first 8 weeks to ensure program acceptability and usability. Finally, the most common negative feedback we received about the interventions was the volume of food—several participants reported having too much food, especially for the meal kit intervention. One participant dropped from the program due to this issue, but others skipped deliveries as needed to reduce volume.

Study generalizability may only be limited to adults 65 and older who have difficulty accessing fresh food. Another limitation was that the food delivery partner required participants to login online to customize their produce prescriptions. As many participants did not have the technical knowledge or resources to do this, our research team had to support phone orders. We used this online ordering system as it was an existing online webstore used by our food delivery partners and had the potential to allow for intervention scalability. We are not aware of any previous food prescription delivery studies that have allowed both customization and delivery, making this a unique aspect of this program. Customization rates, however, were low in the produce prescription condition and lack of internet access was the main reported reason. Future studies should consider using community health workers or other support people to assist with customization; however, this may reduce intervention scalability.

Another limitation of this research is that there is not a standard way to measure F&V usage, and we are not aware of any food prescription studies that have done so previously (outside of measuring consumption). While we relied on self-reported usage, which may be prone to bias, usage was consistent across all groups indicating that redemption (i.e., getting the food into the home) is the most important factor affecting dosage received. A major challenge we faced was that the weekly surveys were not consistently filled out by all participants and there was a much lower response rate in the mobile market group, likely because those who did not go to the market did not complete the survey. Records from our food partners confirmed the delivery rates we saw in the intervention arms. Delivery partner rates were slightly higher (e.g., 92.5% vs. 89.2%) likely due to participants not completing all of their weekly surveys. Delivery rates were not 100% because participants had the option to skip their deliveries if they could not receive a delivery (e.g., they were in the hospital or out of town) or did not want more food. Average participant-reported mobile market redemption rates were higher than partner-reported redemption rates. This difference between self-report and actual redemption in the control arm was likely due to the fact that those who filled out the weekly surveys were more likely to have redeemed their prescription and those who did not were treated as missing.

### 4.2 Conclusion

This study fills a gap in the food prescription literature by examining utilization of food prescription programs. It is also one of the first studies we are aware of to compare the effectiveness of multiple food prescription interventions using a randomized controlled trial design. Overall, it appears that the most important factor for overcoming barriers to food prescription usage in this population was delivery. Future food prescription programs should consider delivery of food prescriptions for older adults with limited food and transportation access. Future research should examine the impact of delivered food prescriptions on diet and health outcomes as well as test these interventions in different populations.

## Data Availability

The raw data supporting the conclusions of this article are available in the Open Science Framework: https://osf.io/e47xs/overview.
