# Supplementary material for: A randomized controlled study comparing three food prescription programs for older adults: program design, implementation, and utilization
Source: Front Public Health. 2026 Sep 24;14:1855988. doi: 10.3389/fpubh.2026.1855988 (PMC13645783; doi:10.3389/fpubh.2026.1855988)
Supplement: Supplementary file 1 [file Table_1.docx]

Supplementary Table 1. Descriptions of Food Prescription Programs Provided to Participants during Recruitment

| Program Arm | Description for Participants |
| --- | --- |
| Delivered Meal Kits | - If you participate in the study, you will be provided with a produce prescription for 12 weeks. - Your produce prescription can be used to get three free healthy meal kits delivered to your home. After you are enrolled in the study, you will complete a baseline phone survey before receiving your first healthy meal kit delivery. - Each week you will get to pick three meal kits from a choice of about 6 meal kit options. - The meal kits are based on recipes created by a nutritionist to help people eat more fruits and vegetables. The weekly meal kits will provide all the ingredients needed to make these recipes. You will receive three meal kits per week for 12 weeks. |
| Delivered Produce Prescription | - If you participate in the study, you will be provided with a produce prescription for 12 weeks. Your produce prescription can be used to have a weekly box of produce delivered to your house. - The contents of the produce box will change weekly and if you want you can choose the produce you will get each week through an online ordering platform. If you make no changes you will receive the standard box. The weekly box will provide 6-12 servings of different fruits and vegetables. You will receive your box for 12 weeks. |
| Mobile Market Produce Prescription | - If you participate in the study, you will be provided with a produce food prescription for 12 weeks. Your produce prescription can be used to get free fresh fruits and vegetables at a local mobile market serving your community. After you are enrolled in the study, you will complete a baseline phone survey before receiving your produce prescription. - Your produce prescription will be a weekly credit to be used at any of the approved mobile market locations in the City of Buffalo, including sites close to the ECMC campus (a full list of sites will be provided during your program orientation). The weekly credit will provide 6-12 servings of fruits and vegetables each week. - You will receive your credit for 12 weeks. Your weekly credit will automatically be applied to an account at the mobile market and you can purchase any produce items you would like. The weekly credits will expire and will not carry over, so you must “use it or lose it” every week to receive the benefit. |
